# *RTEL1* tagging SNPs and haplotypes were associated with glioma development

**DOI:** 10.1186/1746-1596-8-83

**Published:** 2013-05-17

**Authors:** Gang Li, Tianbo Jin, Hongjuan Liang, Zhiguo Zhang, Shiming He, Yanyang Tu, Haixia Yang, Tingting Geng, Guangbin Cui, Chao Chen, Guodong Gao

**Affiliations:** 1 Department of Neurosurgery, Tangdu hospital, the Fourth Military Medical University, Xi’an, 710038, China; 2 National Engineering Research Center for Miniaturized Detection Systems, School of Life Sciences, Northwest University, Xi’an, 710069, China; 3 Department of Clinical Experimental Surgery, Tangdu hospital, the Fourth Military Medical University, Xi’an, 710038, China; 4 Shaanxi Lifegen Co. Ltd, Xi’an, 710069, China; 5 Department of Radiology, Tangdu hospital, the Fourth Military Medical University, Xi’an, 710038, China

**Keywords:** Tagging single nucleotide polymorphism (tSNP), Glioma, *RTEL1*, Haplotype, Case - control studies

## Abstract

**Abstract:**

As glioma ranks as the first most prevalent solid tumors in primary central nervous system, certain single-nucleotide polymorphisms (SNPs) may be related to increased glioma risk, and have implications in carcinogenesis. The present case–control study was carried out to elucidate how common variants contribute to glioma susceptibility. Ten candidate tagging SNPs (tSNPs) were selected from seven genes whose polymorphisms have been proven by classical literatures and reliable databases to be tended to relate with gliomas, and with the minor allele frequency (MAF) > 5% in the HapMap Asian population. The selected tSNPs were genotyped in 629 glioma patients and 645 controls from a Han Chinese population using the multiplexed SNP MassEXTEND assay calibrated. Two significant tSNPs in *RTEL1* gene were observed to be associated with glioma risk (rs6010620, *P* = 0.0016, OR: 1.32, 95% CI: 1.11-1.56; rs2297440, *P* = 0.001, OR: 1.33, 95% CI: 1.12-1.58) by *χ*^2^ test. It was identified the genotype “GG” of rs6010620 acted as the protective genotype for glioma (OR, 0.46; 95% CI, 0.31-0.7; *P* = 0.0002), while the genotype “CC” of rs2297440 as the protective genotype in glioma (OR, 0.47; 95% CI, 0.31-0.71; *P* = 0.0003). Furthermore, haplotype “GCT” in *RTEL1* gene was found to be associated with risk of glioma (OR, 0.7; 95% CI, 0.57-0.86; Fisher’s *P* = 0.0005; Pearson’s *P* = 0.0005), and haplotype “ATT” was detected to be associated with risk of glioma (OR, 1.32; 95% CI, 1.12-1.57; Fisher’s *P* = 0.0013; Pearson’s *P* = 0.0013). Two single variants, the genotypes of “GG” of rs6010620 and “CC” of rs2297440 (rs6010620 and rs2297440) in the *RTEL1* gene, together with two haplotypes of GCT and ATT, were identified to be associated with glioma development. And it might be used to evaluate the glioma development risks to screen the above *RTEL1* tagging SNPs and haplotypes.

**Virtual slides:**

The virtual slides for this article can be found here: http://www.diagnosticpathology.diagnomx.eu/vs/1993021136961998

## Introduction

The overall incidence of brain tumors for benign and malignant tumors combined is 18.71 per 100,000 person-years; 11.52 per 100,000 person-years for benign tumors and 7.19 per 100,000 person-years for malignant tumors [1]. Though the age- standardized incidence recently reported varied greatly than ever, non-malignant tumours still accounted for 66% of all newly diagnosed primary brain tumours with the age-standardized incidence rate of 3.57 per 100,000 person-years, while malignant tumours incidence rate was 1.82 per 100,000 person-years (crude incidence rates were 3.69 and 1.92 per 100,000 respectively) [2]. Gliomas, most aggressive malignant brain tumours (astrocytic, oligodendroglial, oligoastrocytic and ependymal origin), represent 20.8% of all brain tumours [2], and account for almost 80% of primary malignant brain tumors, usually resulting in poor survival compared to other types of brain tumors [3].

Current evidence suggests that inherited risks play a significant role in glioma susceptibility, as with other cancers, and a majority of the inherited risk is due to the co-inheritance of multiple low-risk variants. These variants are commonly seen gene variants and hence can be identified through association studies [4]. The epidemiology of glioma has focused on identifying factors that can be modified to prevent this disease [5-7]. Recently studies of genetic risk factors for brain tumors have expanded to genome-wide association studies, and have focused on identifying germline polymorphisms associated with the risk of glioma as well as using molecular markers to classify glial tumors in more homogenous groups [6,7].

A research group from the University of Texas M.D. Anderson Cancer Center conducted a meta-analysis of two genome-wide association studies (GWAS) by genotyping 550 K tagging single nucleotide polymorphisms (tSNPs) in a total of 1,878 cases and 3,670 controls, with validation in three additional independent series totaling 2,545 cases and 2,953 controls. They identified five risk loci for glioma including rs6010620 intronic to *RTEL1* gene (*P* = 2.52 × 10^-12^) to be associated with glioma risk [6]. Another study in Chinese also identified rs60106203 for glioma risk (*P* = 2.793 × 10^-6^), and the locus also associated with glioblastoma risk (*P* = 3.573 × 10^-7^) [8]. The subsequent study found that rs6010620 was statistically significantly associated with glioma risk in US female population [9]. Recently, a new independent GWAS of glioma using 1,856 cases and 4, 955 controls has found evidence of strong replication for three of the seven previously reported associations at 20q13.33 (*RTEL*), 5p15.33 (*TERT*), and 9p21.3 (*CDKN2BAS*), and consistent association signals for the remaining four at 7p11.2 (*EGFR* both loci), 8q24.21 (*CCDC26*) and 11q23.3 (*PHLDB1*) [7]. These data tend to show that common susceptibility alleles contribute to the risk of developing glioma and provide insight into disease causation of this primary brain tumor.

As the Chinese Han population is by far the population with the largest number in the world, we comprehensively analysized in this study the associations between *RTEL1* genotypes and haplotypes with glioma risk, to uncover how germ-line genetic variants of the *RTEL1* gene play a complex role in the development of glioma, to offer important insights into the etiology of glioma in the certain Chinese Han population.

## Patients and methods

### Study population

A total of 629 patients with glioma, includes well-differentiated pilocytic astrocytoma [World Health Organization (WHO) grade I], low grade ependymomas [WHO grade II], low grade astrocytomas [WHO grade II], low grade oligodendrogliomas [WHO grade II], anaplastic astrocytomas [WHO grade III] and glioblastoma multiforme (GBM) [WHO grade IV] [10], between November 2008 and December 2012 were recruited into an ongoing molecular epidemiological study at the Department of Neurosurgery of the Tangdu Hospital affiliated with The Fourth Military Medical University (FMMU) in Xi’an city, China. All glioma cases had no previous history of other cancers, or prior chemotherapy or radiotherapy. There were no age, sex, or disease stage restrictions for case recruitment. There were no age, sex, or disease stage restrictions for case recruitment. All the slides of glioma tissues were re-evaluated according to WHO classifications [10] by two pathologists, with differences resolved by careful discussion. The median age was 43 years (age range, 1–81). The clinicopathological features and the treatment strategies of all the patients were indicated in Table 1.

**Table 1:** Clinicopathological features of 629 glioma patients

**Clinicopathological features**	**WHO I**	**WHO II**			**WHO III**	**WHO IV**
	**Pilocytic astrocytoma**	**Astrocytoma**	**Oligodendroglioma**	**Ependymoma**	**Anaplastic astrocytoma**	**Glioblastoma multiforme**
**Case Number**						
Total	20	433	24	34	81	37
Male	11	241	13	17	39	21
Female	9	192	11	17	42	16
**Mean age (Age range) (ys)**						
Total	12(2-51)	42(1-81)	46(17-76)	29(1-71)	50(2-81)	47(6-70)
Male	13(3-35)	41(1-81)	53(35-76)	34(5-60)	50(2-73)	48(17-70)
Female	9(2-51)	42(2-79)	41(17-55)	20(1-71)	52(10-81)	44(6-67)
**KPS**						
≥70	19	423	24	33	79	35
<70	1	10	0	1	2	2
**Surgery**						
Gross total resection	20	412	22	32	76	36
Partial resection	0	10	2	1	3	1
Biopsy	0	11	0	1	2	0
**Adjuvant treatment**						
Radiotherapy	0	280	18	17	4	0
Chemotherapy	0	55	1	1	0	0
Radiotherapy and Chemotherapy combination	0	98	5	16	77	37

*KPS* Karnofsky performance score.

A total of 645 healthy unrelated individuals as the controls between June 2010 and August 2012 were recruited from the medical examination center at Tangdu Hospital, for genetic association research of human complex diseases, such as lung cancer, stomach cancer, and glioma. The median age was 45 years (age range 4–83). The detailed recruitment and exclusion criteria were used. Generally, subjects with chronic diseases and conditions involving vital organs (heart, lung, liver, kidney, and brain) and severe endocrinological, metabolic, and nutritional diseases were excluded from this study. The purpose of the above exclusion procedures was to minimize the known environmental and therapeutic factors that influence the variation of human complex diseases.

In our study population, all analyses were restricted to the Han Chinese living in Xi’an city and its surrounding areas. A written informed consent was obtained from all the subjects or their custodians, and we collected all the blood samples from the controls and the patients before chemotherapy or radiotherapy. All specimens were handled and made anonymous according to the ethical and legal standards. The protocol was approved by the Medical Ethics Committee of the Fourth Military Medical University.

### Demographic and clinical data

Demographic and personal data were collected through an in-person interview using a standardized epidemiological questionnaire, including age, sex, ethnicity, residential region, smoking status, alcohol use, education status, and family history of cancer. For patients, detailed clinical information was collected through a medical chart review or consultation with treating physicians. Plasma carcinoembryonic antigen and alpha-fetoprotein were tested in control subjects to make sure they did not have any cancers.

### Blood samples collection, DNA extraction and SNP selection and genotyping

Peripheral blood was taken from the 629 glioma patients and 645 apparently healthy individuals, and from the elbow vein or the head superficial vein, and treated immediately with an anticoagulant containing sodium citrate (22 g/L) and sodium chloride (8.5 g/L). The blood samples were then stored at -70˚C before use. Genomic DNA was isolated from the samples by using an extraction kit (GoldMag, China). DNA concentration and purity were determined by an ultraviolet spectrophotometer (Eppendorf, Hamburg, Germany).

Candidate tSNPs in the seven genes were selected from previously published polymorphisms associated with glioma. Validated tSNPs were selected with a minor allele frequency (MAF) > 5% in the HapMap Asian population. A total of 10 tSNPs were selected for further genotyping. Genomic DNA was extracted from whole blood using the phenol-chloroform extraction method. DNA concentration was measured by spectrometry (DU530 UV/VIS spectrophotometer, Beckman Instruments, Fullerton, CA, USA). A multiplexed SNP MassEXTEND assay was designed with the Sequenom MassARRAY Assay Design 3.0 Software [11]. SNP genotyping was performed using the Sequenom MassARRAY RS1000 with standard protocol recommended by the manufacturer [11]. Data management and analyses were performed using Sequenom Typer 4.0 software as previously described [11,12].

### Statistical analysis

Statistical analyses were performed using Microsoft Excel and SPSS 16.0 statistical packages (SPSS, Chicago, IL). All *P* values in this study were two-sided. A *P* ≤ 0.05 was considered the threshold of statistical significance. Genotypic frequencies in control subjects for each tSNP were tested for departure from Hardy–Weinberg equilibrium (HWE) using an exact test. Genotype frequencies and allele frequencies of glioma patients and control subjects were compared using the *χ*^2^ test [13]. Odds ratios (ORs) and 95% confidence intervals (CIs) were calculated by unconditional logistic regression analysis with adjustment for age and sex [14]. We did not divide subjects into subgroups because of limited sample size. The possibility of sex differences as a source of population sub-structure was evaluated by a genotype test for each tSNP in male and female controls, and the number of significant results at the 5% level was compared with the number expected by the *χ*^2^ test. We did not detect population stratification because all participants’ ethnicity was Han Chinese. The four genetic models (dominant, recessive, additive and genotypic) were applied by PLINK software (http://pngu. mgh. harvard.edu/purcell/plink/) to assess the association of single tSNP with the risk of glioma. ORs and 95% CIs were calculated by unconditional logistic regression analysis adjusted for age and sex [14,15].

We used the Haploview software package (version 4.2) and SHEsis software platform (http://www.nhgg.org/analysis/) for analyses of linkage disequilibrium, haplotype construction, and genetic association at polymorphism loci [16,17] ORs and 95% CIs were calculated by unconditional logistic regression analysis with adjustment for age and gender [14]. Additionally, the likelihood ratio test was performed to determine the genotype frequencies among various grade groups. The *χ*2 test was also used for comparison of categorical variables. A *P* value of <0.05 (two-tailed) was considered statistically significant.

## Results

A total of 629 cases (310 male, 319 female; median age at diagnosis 41 ± 18 yrs) and 645 controls (329 male, 316 female; median age at 45 ± 12 yrs) were included in the current study. Basic characteristics of the cases are listed in Table 1 including gender, age, and pathology. As listed in Table 2, a multiplexed SNP MassEXTEND assay was designed with Sequenom MassARRAY Assay Design 3.0 Software. Ten SNPs of seven candidate genes were genotyped in glioma patients and the control group, the average tSNPs call rate was 99.6% in cases and controls, and all of the tested tSNPs are in Hardy–Weinberg equilibrium (HWE) in the control population of this study (Table 3). Two significant tSNPs in the *RTEL1* gene were observed to be associated with glioma risk at a 5% level (rs6010620, *P* = 0.0016, OR: 1.32, 95% CI: 1.11-1.56; rs2297440, *P* = 0.001, OR: 1.33, 95% CI: 1.12-1.58) by *χ*^2^ test.

**Table 2:** Primers used in the study

**SNP_ID**		**1st-PCR primer sequences**	**2nd-PCR primer sequences**	**UEP sequences**
rs2992	G/A	ACGTTGGATGTCAAGTATCTGCTCTGTGGG	ACGTTGGATGACTGGGTGCATCCTGAGAG	cgatAGCAGGGGTGACGTATGTAGAA
rs12022378	C/T	ACGTTGGATGAGATGCCTGGACCAGCTCT	ACGTTGGATGCAATTACAGCCCACCTCTTG	cctaaAGCCCACCTCTTGCATCGT
rs12917	T/C	ACGTTGGATGCGAGGCTATCGAAGAGTTCC	ACGTTGGATGAGATGGCTTAGTTACCGACC	gctaGAAAACGGGATGGTGAA
rs12645561	T/C	ACGTTGGATGTTACAGTTCTCTTTCACAG	ACGTTGGATGGCAGAGCCTAGTTTCATGAC	TTGCTCATTACTGTAAGAAATAATAC
rs7003908	C/A	ACGTTGGATGGGGGAGAAAATATTCCTGTT	ACGTTGGATGTCCTACCTCACGAACTCAGC	AGCAATTGCCTAAGAGTC
rs6010620	G/A	ACGTTGGATGGCCTGTTTTCCCTTTTTGAG	ACGTTGGATGCCTCTCAACATCTCAGCAAC	tGATCATGCAAAGCAGG
rs2297440	T/C	ACGTTGGATGACGAGGTCTGGTGGCACAT	ACGTTGGATGCACTGTCCTTTGCGTCCTC	gtTCCTCCCTCACCAGC
rs4809324	C/T	ACGTTGGATGGAGAAGTCAAGTGACATCAG	ACGTTGGATGAGCCGGTGCACAGATTCCAA	gagggCAAGGGCCTGGAATCTGT
rs3770502	A/G	ACGTTGGATGCTATATGGGTGCAGATGCAG	ACGTTGGATGACAGGCGTGAACCACTGTA	ACCCGGCCCCTCCAC
rs9288516	A/T	ACGTTGGATGACAGGCCAAGGGCAATAATC	ACGTTGGATGGCTTCCTAAGATTTCCTATTC	CATTTCAAAAGAAATGGAGAAT

*UEP* Unextended mini-sequencing primer.

**Table 3:** Tagging SNPs information that were examined

**SNP No.**	**Gene**	**chr**	**Position**	**MA**	**MAF (CHB)**	**MAF**		**HWE *P***	***P***	**OR (95%CI)**	***P adj.***	**Genotype rate**
						**Case**	**Control**					
rs12022378	*AP4B1*	chr1	114448389	C	0.411	0.360	0.393	0.4943	0.0889	1.15(0.98-1.35)	0.889	99.34
rs12917	*MGMT*	chr10	131506283	T	0.31	0.105	0.082	0.2082	0.0497	0.76(0.58-1)	0.497	99.67
rs12645561	*NEIL3*	chr4	178260872	T	0.114	0.275	0.268	0.1388	0.6903	0.97(0.81-1.15)	6.903	99.34
rs7003908	*PRKDC*	chr8	48770702	C	0.283	0.236	0.209	0.1246	0.1074	0.86(0.71-1.03)	1.074	99.83
rs6010620	*RTEL1*	chr20	62309839	G	0.393	0.269	0.327	0.194	0.0016	1.32(1.11-1.56)	0.016	99.83
rs2297440	*RTEL1*	chr20	62312299	C	0.226	0.266	0.325	0.3127	0.001	1.33(1.12-1.58)	0.01	100
rs4809324	*RTEL1*	chr20	62318220	C	0.12	0.114	0.120	0.6994	0.6328	1.06(0.83-1.35)	6.328	99.67
rs2992	*UBXN6*	chr19	4443046	A	0.295	0.436	0.433	0.4229	0.8701	0.99(0.84-1.16)	8.701	99.67
rs3770502	*XRCC5*	chr2	217045059	A	0.208	0.148	0.160	0.7558	0.4221	1.09(0.88-1.36)	4.221	99.83
rs9288516	*XRCC5*	chr2	217053264	A	0.489	0.480	0.450	0.4774	0.1288	0.89(0.76-1.04)	1.288	99.67

Association results between tSNP genotypes and the risk of glioma were listed in Table 4. We found that the genotype “GG” of rs6010620 as the protective genotype for glioma (OR, 0.46; 95% CI, 0.31-0.7; *P* = 0.0002), and the genotype “CC” of rs2297440 as the protective genotype in glioma (OR, 0.47; 95% CI, 0.31-0.71; *P* = 0.0003).

**Table 4:** Association between tSNP genotypes and the risk of glioma

**SNP ID**	**Genotype**	**No. (frequency)**		**Logistic regression**	
		**Case**	**Control**	**OR (95% CI)**	***P *value**
rs12022378	CC	114(18.2)	87(13.6)	0.71(0.51-0.99)	0.0418
rs12022378	CT	265(42.2)	288(44.9)	1.01(0.8-1.29)	0.9127
rs12022378	TT	249(39.6)	267(41.6)	1(referent)	
rs12917	TT	6(1)	10(1.6)	1.7(0.61-4.72)	0.3011
rs12917	TC	91(14.5)	115(17.9)	1.29(0.96-1.74)	0.0956
rs12917	CC	530(84.5)	519(80.6)	1(referent)	
rs12645561	TT	43(6.8)	56(8.7)	1.26(0.83-1.93)	0.2784
rs12645561	TC	251(40)	241(37.6)	0.93(0.74-1.18)	0.5537
rs12645561	CC	334(53.2)	344(53.7)	1(referent)	
rs7003908	CC	31(4.9)	43(6.7)	1.44(0.89-2.33)	0.1387
rs7003908	CA	201(32)	217(33.7)	1.12(0.88-1.42)	0.3536
rs7003908	AA	397(63.1)	383(59.6)	1(referent)	
rs6010620	GG	75(11.9)	40(6.2)	0.46(0.31-0.7)	0.0002
rs6010620	GA	261(41.5)	267(41.5)	0.89(0.71-1.12)	0.3212
rs6010620	AA	293(46.6)	337(52.3)	1(referent)	
rs2297440	CC	73(11.6)	40(6.2)	0.47(0.31-0.71)	0.0003
rs2297440	CT	263(41.8)	263(40.8)	0.86(0.68-1.08)	0.1902
rs2297440	TT	293(46.6)	342(53)	1(referent)	
rs4809324	CC	11(1.8)	7(1.1)	0.62(0.24-1.6)	0.3162
rs4809324	CT	129(20.5)	133(20.7)	1(0.76-1.31)	0.9901
rs4809324	TT	488(77.7)	504(78.3)	1(referent)	
rs2992	AA	126(20.1)	117(18.2)	0.98(0.71-1.35)	0.9003
rs2992	AG	292(46.5)	328(50.9)	1.19(0.92-1.52)	0.1822
rs2992	GG	210(33.4)	199(30.9)	1(referent)	
rs3770502	AA	12(1.9)	15(2.3)	1.18(0.54-2.54)	0.6809
rs3770502	AG	177(28.1)	161(25)	0.86(0.67-1.1)	0.2198
rs3770502	GG	440(70)	468(72.7)	1(referent)	
rs9288516	AA	128(20.4)	153(23.8)	1.28(0.93-1.74)	0.1249
rs9288516	AT	308(49.1)	312(48.4)	1.08(0.84-1.4)	0.5539
rs9288516	TT	191(30.5)	179(27.8)	1(referent)	

*OR* odd ratio, *CI* confidence interval.

We assumed that the minor allele of each tSNP was a risk allele compared to the wild type allele. Four tSNPs were detected to be associated with glioma by model association analyses including rs6010620 and rs2297440 in the *RTEL1* gene, rs12022378 in the *DCLRE1B* gene, and rs12917 in the *MGMT* gene (Table 5). We observed two tSNPs in *RTEL1* gene to be associated with the risk of glioma by recessive model (rs6010620, OR, 2.09; 95% CI, 1.39-3.13; *P* = 0.0004, and rs2297440, OR, 2.02; 95% CI, 1.35-3.04; *P* = 0.0007). Rs12022378 in the *DCLRE1B* gene was also found by recessive model associated with glioma risk (OR, 1.42; 95% CI, 1.05-1.93; *P* = 0.0246). Rs6010620 and rs2297440 were also detected by Dominant Model with increased risk of glioma (rs6010620, OR, 1.26; 95% CI, 1.01-1.57; *P* = 0.041, and rs2297440, OR, 1.3; 95% CI, 1.04-1.62; *P* = 0.022). Another SNP, rs12917 in the *MGMT* gene, was associated with decreased glioma risk by recessive model analysis (OR, 0.73; 95% CI, 0.54-0.98; *P* = 0.036). Rs6010620, rs2297440 and rs12917 were also found to be associated with glioma risk by additive model analyses (rs6010620, OR, 1.32; 95% CI, 1.11-1.57; *P* = 0.0015, rs2297440, OR, 1.34; 95% CI, 1.12-1.59; *P* = 0.001 and rs12917, OR, 0.75; 95% CI, 0.58-0.99; *P* = 0.038). Genotypic model analyses results shown that three tSNPs were significant to be associated with glioma risk (rs6010620, OR, 1.48; 95% CI, 1. 2-1.83; *P* = 0.0002, rs2297440, OR, 1.47; 95% CI, 1.19-1.82; *P* = 0.000, and rs12022378, OR, 1.19; 95% CI, 1.0-1.4; *P* = 0.043).

**Table 5:** Association between tSNPs and the risk of glioma and their heterozygote and homozygote odds ratios, per allele odds ratios and confidence intervals

**SNP No.**	**Dominant model**				**Recessive model**				**Additive model**				**Genotypic model**			
	**OR**	**95% CI**		***P*-value**	**OR**	**95% CI**		***P*-value**	**OR**	**95% CI**		***P *value**	**OR**	**95% CI**		***P *value**
rs12022378	1.08	0.86	1.36	0.4966	1.42	1.05	1.93	0.0246	1.14	0.97	1.33	0.1031	1.19	1.01	1.40	0.0426
rs2992	0.90	0.71	1.14	0.3963	1.14	0.86	1.51	0.3714	0.99	0.85	1.16	0.9496	1.02	0.87	1.19	0.8340
rs12917	0.73	0.54	0.98	0.0359	0.70	0.25	1.94	0.4882	0.75	0.58	0.98	0.0383	0.81	0.49	1.36	0.4362
rs12645561	1.02	0.82	1.28	0.8315	0.74	0.49	1.12	0.1499	0.96	0.81	1.15	0.6584	0.87	0.70	1.08	0.2142
rs7003908	0.87	0.69	1.10	0.2372	0.74	0.46	1.19	0.2102	0.87	0.73	1.05	0.1492	0.84	0.66	1.08	0.1684
rs6010620	1.26	1.01	1.57	0.0410	2.09	1.39	3.13	0.0004	1.32	1.11	1.57	0.0015	1.48	1.20	1.83	0.0002
rs2297440	1.30	1.04	1.62	0.0221	2.02	1.35	3.04	0.0007	1.34	1.12	1.59	0.0010	1.47	1.19	1.82	0.0003
rs4809324	1.03	0.79	1.34	0.8386	1.76	0.67	4.60	0.2490	1.06	0.83	1.36	0.6257	1.33	0.82	2.14	0.2513
rs3770502	1.16	0.91	1.48	0.2314	0.79	0.37	1.72	0.5557	1.11	0.89	1.38	0.3668	0.91	0.62	1.34	0.6410
rs9288516	0.90	0.70	1.14	0.3803	0.84	0.64	1.10	0.1972	0.90	0.77	1.05	0.1896	0.90	0.77	1.05	0.1789

*OR* odd ratio, *CI* confidence interval.

Only one block was detected in *RTEL1* gene by haplotype analysis. Global result for the block was: total case = 1286, total control =1256, global *χ*^2^ = 13.0855 while df = 2, Fisher’s *P* value = 0.0015, and Pearson’s *P* value = 0.0014. The results of the association between the *RTEL1* gene haplotypes and the risk of glioma are listed in Table 6. Haplotype “GCT” in *RTEL1* gene was found to be associated with risk of glioma (OR, 0.7; 95% CI, 0.57-0.86; Fisher’s *P* = 0.0005; Pearson’s *P* = 0.0005). Haplotype “ATT” was found to be associated with risk of glioma (OR, 1.32; 95% CI, 1.12-1.57; Fisher’s *P* = 0.0013; Pearson’s *P* = 0.0013).

**Table 6:** **Haplotype frequencies of *RTEL1 *gene and association with risk of glioma in cases and controls**

**Haplotype**	**Freq(case)**	**Freq(ctrl)**	**Chi2**	**Fisher’s *P***	**Pearson’s *P***	**Odds ratio**	**[95% CI]**
A T T	0.7294	0.6728	10.3722	0.0013	0.0013	1.32	[1.12,1.57]
G C C	0.1135	0.1194	0.1959	0.6581	0.6581	0.95	[0.74,1.21]
G C T	0.1524	0.2054	11.9905	0.0005	0.0005	0.70	[0.57,0.86]

Note: Loci chosen for hap-analysis: rs6010620, rs2297440 and rs4809324 (RTEL1); *OR* odd ratio, *CI* confidence interval.

Furthermore, the associations between different clinicopatholiogical features and genotype frequency of GG in rs6010620, together with CC in rs2297440 in glioma patients (*n* = 75, 73, respectively) were analyzed. GG frequency in various grade goups were determined, being 16.0% (12/75), 46.7% (35/75), 16.0% (12/75), and 21.3% (16/75), respectively, in grade I,II,III, and IV group (*P* > 0.05), and CC frequency in various grade goups were determined as 14.7% (11/73), 56.2% (35/73), 17.8% (13/73), and 19.2% (14/73), respectively, in grade I, II, III, and IV group (P >0.05) (Table 7). No significant association was found between genotype frequency of GG or CC, and other parameters including WHO grading, gender, age at diagnosis, or Karnofsky performance score (KPS).

**Table 7:** **Associations between different clinicopatholiogical features and genotype frequency of GG in rs6010620, and CC in rs2297440 of *RTEL1 *gene in glioma patients (*n*** **= 75, 73, respectively)**

**Clinicopatholiogical features**	**GG frequency**	***P *value**	**CC frequency**	***P *value**
	***n *(%)**		***n *(%)**	
**WHO grade**				
I	12 (16.0)	>0.05	11 (14.7)	>0.05
II	35 (46.7)		35 (56.2)	
III	12 (16.0)		13 (17.8)	
IV	16 (21.3)		14 (19.2)	
**Age**				
≥40	36 (48.0)	NS	40 (54.8)	NS
<40	39 (52.0)		33 (45.2)	
**Gender**				
Male	30 (40.0)	NS	31 (42.5)	NS
Female	45 (60.0)		42 (57.5)	
**KPS**				
≥70	72 (96.0)	>0.05	70 (95.9)	>0.05
<70	3 (4.0)		3 (4.1)	

*NS* not significant, *KPS* Karnofsky performance score.

## Discussion

As known, biomarker detection and screening is an emerging field for oncology [18-23]. Especially for gliomas considerable progresses have been made in identifying, characterizing, and attempting to apply molecular markers, e.g. in the previous study, we initially found the increased expression of miR-372 in glioma tissues was significantly correlated with advanced tumor progression and aggressive clinicopathological features [24]. And subsequently a series of have determined the associations between lots of SNPs in *ABCB* 1, *NR* 1/2, *VEGFR* 3, etc. and therapy outcome [25-27].

In this case–control study in Han Chinese population, we found two susceptibility tSNPs in *RTEL1* gene that were associated with increased risk of glioma (rs6010620 and rs2297440). The genotype “GG” of rs6010620 was the protective genotype for glioma, and the genotype “CC” of rs2297440 was the protective genotype in glioma. We also observed in the *RTEL1* gene a haplotype “GCT” that was associated with a decreased the risk and a haplotype of “ATT” with an increased risk of developing glioma.

As described initially, however, we failed to determine the associations between genotype frequency of GG or CC, and other parameters including WHO grading, gender, age at diagnosis, or KPS status. Additionally, we also tried to elucidate the relationship of genotype frequency of GG or CC, with overall surviaval (OS) in the correlated patients. During the follow-up period, only 11 of the patients with genotype of GG or CC had died [6 (8%) from the 75 patients with genotype GG, and 5 (6.8%) from the 73 patients with genotype CC], and most of the patients are alive and are being traced continuously.

Albeit the correlated survival data in the present study are still accumulating, our findings in this study have provided new evidence for the association between common SNPs (or haplotypes) and the risk of glioma in the Chinese Han population, suggesting an important determinant of glioma development by *RTEL1* gene. *RTEL1* gene locates in 20q13.3 with the length of 40.889 kb, including 40 exons. Known functions of *RTEL1* include nucleic acid binding, ATP-dependent DNA helicase activity, DNA repair, apoptosis and anti-apoptosis, and so on. Previous study proposed that *RTEL1* maintains genomic stability by suppressing homologous recombination [28,29], and implements the second level of meiotic crossover control by promoting non-crossovers [30,31]. A recent review point out that *RTEL1* was an essential helicase for telomere maintenance and the regulation of homologous recombination [32,33]. *RTEL1* didn’t involve any KEGG pathway (http://www. genome.jp/kegg/) so far. The frequencies of rs6010620 risk genotypes were highly correlated with high-grade disease (*P* < 0.001), indicating that genetic variations at the locus has subtype-specific effects on the risk of developing glioma [34]. *RTEL1* gene was over expressed in human gastrointestinal tract tumors [35]. Polymorphism in the *RTEL1* gene was associated with glioblastoma survival [36].

Some limitations are inherent in this case–control study and must be noted here. Glioma patients were not sub-grouped by age or gender, and gender-specific significant variants were not tested. We selected tSNPs with frequencies higher than 5% in HapMap Asian populations to affirm the statistical power was large enough for analyzing data. We also designed a haplotype-based study to ensure sufficiently high power to detect the risk of glioma associated with candidate tSNPs. Another potential concern was population admixture, which is a known confounding factor for association analysis and may also result in inflated type-I error (false positive). In this study, glioma patients and controls were used in the same hospital to avoid the possibility that one may have a more pronounced selection bias. However, this bias is unlikely to be of significance because they did not differ in the distributions of demographic variable and genotype frequencies. We limited all subjects’ ethnicity to Han Chinese, and a living area to Xi’an City and its surrounding area, thus there is no substantial population admixture in our study populations.

In the upcoming studies, our team will go on to follow up the subjects recruited into the present study, and carry out additional research with larger subject numbers and grade types to further characterize the relationship among grades within the individuals, clinical features and the mentioned *RTEL1* tagging SNPs & haplotypes. Furthermore, to elucidate the role of the *RTEL1* gene in gliomagenesis, serum *RTEL1* expression levels between different mutations or haplotype groups will be compared. And, we will also investigate the association between germline *RTEL1* variants and somatic *RTEL1* mutations, and the relationship between serum *RTEL1* expression and somatic *RTEL1* expression in the same glioma subjects.

## Conclusion

In conclusion, our comprehensive analysis of tSNPs suggests that the genotypes of “GG” of rs6010620 and “CC” of rs2297440 (rs6010620 and rs2297440) in the *RTEL1* gene, together with two haplotypes of GCT and ATT, were identified to be associated with glioma development. And it might be used to evaluate the glioma development risks to screen the above *RTEL1* tagging SNPs and haplotypes.

## Competing interests

The authors declare that they have no competing interests.

## Authors’ contributions

CC and GG were the overall principle investigator of the study who designed the study and were responsible for study design, and interpreted the results. GL and TJ participated in the design and coordination, performed the molecular genetic evaluation, and drafted the manuscript. And ZZ, GC, HY, together with TG performed the statistical analysis, and joined into drafting the manuscript. All the patients were followed up by YT, SH and HL. GL, CC and GG all contributed to improving the draft of the manuscript. All authors have read and approved the final manuscript.
